# Draft Genome Sequence of *Rhodococcus rhodochrous* Strain ATCC 21198

**DOI:** 10.1128/genomeA.00054-14

**Published:** 2014-02-13

**Authors:** Sara A. Shields-Menard, Steven D. Brown, Dawn M. Klingeman, Karl Indest, Dawn Hancock, Jayani J. Wewalwela, W. Todd French, Janet R. Donaldson

**Affiliations:** a Department of Biological Sciences, Mississippi State University, Mississippi State, Mississippi, USA; b Biosciences Division, Oak Ridge National Laboratory, Oak Ridge, Tennessee, USA; c BioEnergy Science Center, Oak Ridge National Laboratory, Oak Ridge, Tennessee, USA; d U.S. Army Engineer Research and Development Center, Environmental Laboratory, Vicksburg, Mississippi, USA; e Dave C. Swalm School of Chemical Engineering, Mississippi State University, Mississippi State, Mississippi, USA

## Abstract

*Rhodococcus rhodochrous* is a Gram-positive red-pigmented bacterium commonly found in the soil. The draft genome sequence for *R. rhodochrous* strain ATCC 21198 is presented here to provide genetic data for a better understanding of its lipid-accumulating capabilities.

## GENOME ANNOUNCEMENT

*Rhodococcus rhodochrous* is a red pigment-forming Gram-positive bacterium belonging to the *Actinobacteria* phylum and the *Nocardiaceae* family. This metabolically diverse bacterium has been most notably used for the industrial production of acrylamide from acrylonitrile and in the bioremediation of hydrocarbons, polychlorinated biphenyls, and other aromatic compounds (1–4). Other members of the *Actinobacteria*, such as *Streptomyces*, *Nocardia*, *Rhodococcus*, and *Gordonia*, have been shown to synthesize and accumulate triacylglycerols (TAGs) (5). The potential for *R. rhodochrous* to utilize a variety of carbon sources for the accumulation of TAGs is of interest due to the increasing global need for alternative biofuels. Despite the widespread use of *R. rhodochrous* in degradation research, the understanding of its ability to lipid accumulation is limited.

A draft genome sequence was generated for strain ATCC 21198 to develop a better understanding of the lipid-accumulating capabilities of *R. rhodochrous*. The isolate *R. rhodochrous* ATCC 21198 was purchased from American Type Culture Collection (ATCC). Genomic DNA was isolated from frozen cell pellets using the Mo-Bio PowerSoil DNA isolation kit (Mo-Bio Laboratories, Inc., Carlsbad, CA), modified by the addition of lysozyme.

Sequence data were generated using an Illumina MiSeq instrument (6) according to the manufacturer’s instructions, using a paired-end approach with an approximate insert library size of 400 bp and read lengths of 250 bp. The CLC Genomics Workbench (version 6.5) was used to trim and filter reads for quality sequence data and subsequent assembly. The draft genome sequence for *R. rhodochrous* ATCC 21198 is represented by 161 DNA contigs, with an estimated genome size of ~6.4 Mb and a G+C DNA content of 70.2%. The average sequence depth coverage across the genome was ~214 times the genome size, which was annotated as described previously (7) for 6,039 predicted protein-coding gene models.

In response to an environment with little nitrogen and excess carbon, oleaginous microbes will store carbon as lipids (5, 8). *Rhodococcus* species have been shown to produce large amounts of lipids in the form of TAGs due to the high activity of the enzyme acyl-coenzyme A (CoA):diacylglycerol acyltransferase (DGAT) (9). In this draft sequence of *R. rhodochrous*, there are several predicted wax ester synthase/diacylglycerol acyltransferase genes showing sequence identity to other *Rhodococcus* species. Other putative fatty acid and TAG synthesis genes were also discovered in the *R. rhodochrous* genome, such as acetyl-CoA carboxylase, acyl carrier proteins, (1-acyl-G3P) acyltransferase, glycerol kinase, and glycerol-3-phosphate dehydrogenase (10). Several cytochrome P450-like enzymes were predicted in the genome of *R. rhodochrous*, as well as the associated genes flavodoxin and feredoxin. Other predicted genes related to degradation mechanisms include oxygenases, dioxygenases, and a putative aromatic degradation protein. The *R. rhodochrous* draft genome offers insight into the potential metabolic capabilities of this organism and will facilitate further studies.

### Nucleotide sequence accession numbers.

This draft genome sequence has been deposited in GenBank under the accession no. AZHI00000000. The version described in this paper is version AZHI01000000.
